# NMR and structural data for Connexin 32 and Connexin 26 N-terminal peptides

**DOI:** 10.1016/j.dib.2016.08.044

**Published:** 2016-09-12

**Authors:** Yuksel Batir, Thaddeus A. Bargiello, Terry L. Dowd

**Affiliations:** a Department of Chemistry, Brooklyn College, Brooklyn, N.Y. 11210, USA; b Ph.D. Program in Chemistry and Biochemistry, The Graduate Center of the City University of New York, New York, NY 10016, USA; c Department of Neuroscience, Albert Einstein College of Medicine, Bronx, New York 10461, USA

## Abstract

In this article we present ^1^H and ^13^C chemical shift assignments, secondary structural propensity data and normalized temperature coefficient data for N-terminal peptides of Connexin 26 (Cx26), Cx26G12R and Cx32G12R mutants seen in syndromic deafness and Charcot Marie Tooth Disease respectively, published in “Structural Studies of N-Terminal Mutants of Connexin 26 and Connexin 32 Using 1H NMR Spectroscopy” (Y. Batir, T.A. Bargiello, T.L. Dowd, 2016) [1]. The mutation G12R affects the structure of both Cx26 and Cx32 peptides differently. We present data from secondary structure propensity chemical shift analysis which calculates a secondary structure propensity (SSP) score for both disordered or folded peptides and proteins using the difference between the ^13^C secondary chemical shifts of the Cα and Cβ protons. This data supplements the calculated NMR structures from NOESY data [1]. We present and compare the SSP data for the Cx26 vs Cx26G12R peptides and the Cx32 and Cx32G12R peptides. In addition, we present plots of temperature coefficients obtained for Cx26, Cx26G12R and Cx32G12R peptides collected previously [1] and normalized to their random coil temperature coefficients, “Random coil 1H chemical shifts obtained as a function of temperature and trifluoroethanol concentration for the peptide series GGXGG” (G. Merutka, H.J. Dyson, P.E. Wright, 1995) [2]. Reductions in these normalized temperature coefficients are directly observable for residues in different segments of the peptide and this data informs on solvent accessibility of the NH protons and NH protons which may be more constrained due to the formation of H bonds.

**Specifications Table**

**Table:** 

Subject area	Biochemistry, Neuroscience
More specific subject area	NMR structural analysis
Type of data	Tables, graphs
How data was acquired	Varian INOVA 600 MHz spectrometer equipped with a cryoprobe.
Data format	Raw, analyzed.
Experimental factors	22 amino acid peptides of Connexin 26 and Connexin 32 and G12R mutants peptides (1.0–1.2 mM) were dissolved in 0.1 M KCl, 10% ^2^H_2_O/90% H_2_O at pH 7.0 and 15 ° C.
Experimental features	Long (80 ms) and short (15 ms) mixing time ^1^H TOCSY spectra and a NOESY spectrum (225 ms mixing time) were collected along with a natural abundance ^13^C HSQC spectrum on a Varian INOVA 600 MHz spectrometer equipped with a cryoprobe as described in [1]
Data source location	Brooklyn, NY
Data accessibility	Data is within this article.

**Value of the data**

• The ^1^H and ^13^C chemical shift data may be helpful to other NMR spectroscopists working on high resolution structures of different Connexin peptides or peptides with similar amino acid sequences.
• Both the ^1^H and ^13^C chemical shift assignments for the Connexin peptides in this study, along with their high resolution structures [1], are useful for developing and testing theoretical methods that predict secondary structure and flexibility using chemical shift data alone. This is extremely useful for the many disordered proteins or disordered regions in proteins which do not give many NOES.
• The temperature coefficient data is useful to obtain information on structure and flexibility to supplement NMR structural data. This data is a good source for comparison of similar data obtained from other protein or peptide structural studies.

## 1 Data

**Fig. 1:**
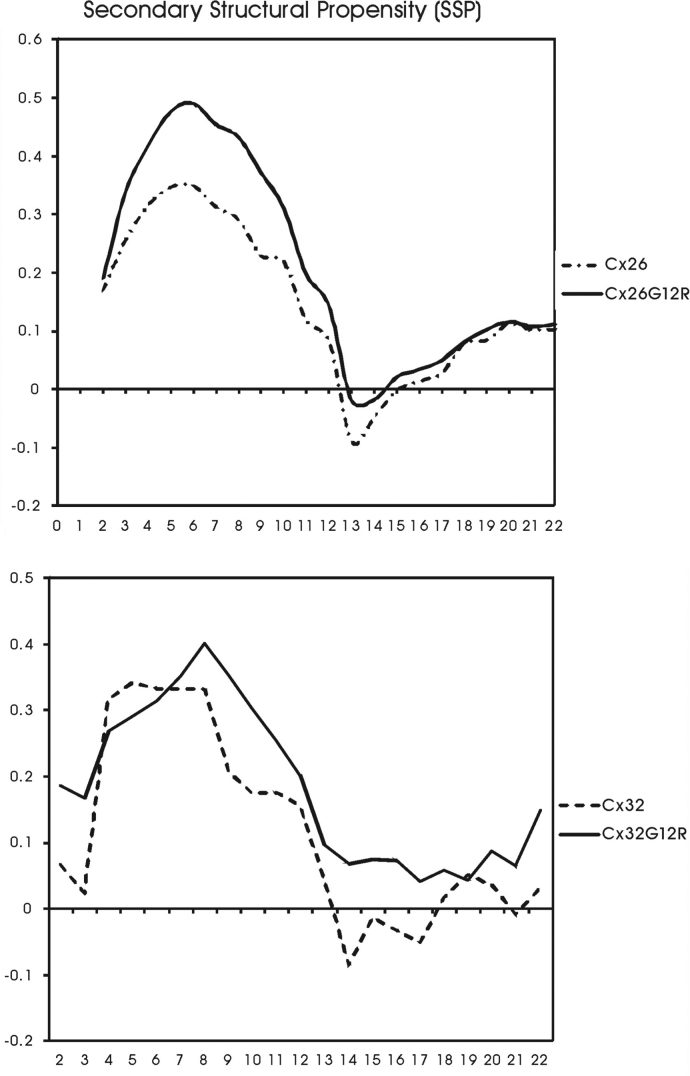
Secondary structure propensity (SSP) scores for Cx26 and Cx26G12R peptides (top) and Cx32 and Cx32G12R peptides (bottom) calculated from ^13^Cα and ^13^Cβ chemical shifts.

**Fig. 2:**
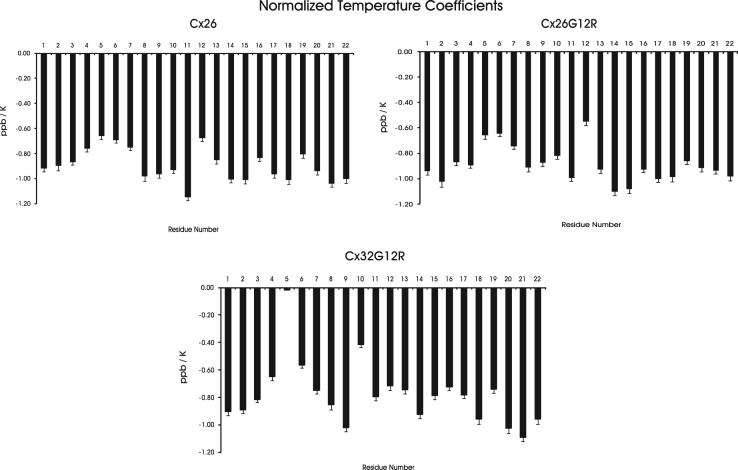
Plots of experimentally measured temperature coefficients for each residue normalized to its random coil temperature coefficient for Cx26, Cx26G12R and Cx32G12R peptides.

Table 1, Table 2, Table 3 contain ^1^H and ^13^C chemical shift assignments for the Cx26, Cx26G12R and Cx32G12R peptides respectively. Fig. 1 is a plot of secondary structural propensity along the sequence of the peptide for Cx26 and Cx26G12R, and Cx32 and Cx32G12R peptides. A positive score indicates helical propensity, a negative score indicates β sheet propensity and values close to zero indicate random coil structure. Fig. 2 is a plot of temperature coefficients for NH protons normalized to their fully solvent exposed, random coil values for Cx26, Cx26G12R, Cx32G12R peptides.

## 2 Experimental design, materials and methods

N-terminal peptides of Cx26 (MDWGTLQTILGGVNKHSTSIGK), Cx26 mutant G12R and Cx32 mutant G12R (MNWTGLYTLLSRVNRHSTAIGR) were synthesized by the New England Peptide Co. using Fmoc chemistry [3]. Samples were prepared and data collected and analyzed as described previously with all peptides dissolved in 0.1 M KCl in 10% ^2^H_2_O/90% H_2_O at pH 7.0 with 100 mM 3-(trimethylsilyl)propionic acid (TSP) used as a chemical shift reference.

Long (80 ms) and short (15 ms) mixing time TOCSY spectra and a NOESY spectrum (225 ms mixing time) were collected to assign all protons. A natural abundance ^13^C HSQC spectrum was collected to assign all Cα and Cβ carbons for each amino acid in all peptides. Data were processed using NMRPipe [4] and analyzed using NMRView [5]. Resonances were assigned using standard procedures previously described [6]. The resonance assignments for the Connexin 26, Cx26G12R and Cx32G12R peptides are presented in Table 1, Table 2, Table 3.

Secondary ^13^C chemical shifts (*Δδ*=*δ*_observed_−*δ*_random coil_) are used as a measure of secondary structure. Secondary structural propensity reports the difference between secondary shifts (*Δδ*^13^C_α_−*Δδ*^13^C_β_) and is also a measure of secondary structure with the advantage of canceling out chemical shift referencing errors and because both α helical and β sheet propensities can be well distinguished. The secondary structural propensity scores (SSP) for each peptide were calculated using the SSP program [7] and the ^13^C_α_ and ^13^C_β_ chemical shifts. The secondary structural propensity plots (SSP) in Fig. 1 show the plots of secondary structural propensity scores for the Cx26, Cx26G12R, Cx32 and Cx32G12R N-terminal peptides along the sequence of the peptides. The positive values indicate helical structure and negative values indicate β sheets with values of approximately 2 representing fully formed structure while lower values indicate partial structure or propensity and more flexibility. Values close to zero indicate unstructured, more flexible regions. Fig. 1 shows the comparison between Cx26G12R and Cx26 and values of secondary structural propensity. Although the Cx26G12R peptide had a slightly higher structural propensity the plots were similar showing partial helical structure from residues 3–11 with very low ssp values from residues 11–16 for both N-termini supporting flexible turn regions or unstructured regions in this area as seen in the NMR structures [1]. The remainder of the peptide from residues 16–22 has low ssp values also indicating an unstructured region which is in agreement with calculated NMR structures [1]. The Cx26G12R peptide had a higher structural propensity but did not have as many NOEs and did not have well defined helical structure [1] suggesting it is a dynamic, flexible structure. The plot comparing the Cx32 and Cx32G12R secondary structural propensity values shows helical propensity for both peptides within residues 3-8 with a greater propensity for helicity for Cx32G12R from residue 9–13 as compared to Cx32. This shows less flexibility within residues 9–13 for Cx32G12R, in agreement with the NMR calculated structure which shows a more constricted helical turn in this region. The NMR structure of Cx32 has a more open turn in this region, in agreement with the low SSP values. Thus N-termini of Cx26, Cx26G12R and Cx32G12R show values very close to zero within the turn regions (residues 13–16) supporting a flexible turn, as seen in the NMR structures, while this region in Cx32G12R is a little higher and suggests more structure and less flexibility. This data agrees with the calculated structures from NMR data we have presented in the manuscript [1].

Long mixing time (80 ms) ^1^H TOCSY spectra were collected at 278, 283, 288 and 293 K for the temperature coefficient data. The NH proton ppm values were recorded at each temperature for all amino acids in the peptides. Temperature coefficients were calculated from the slope of plots of NH chemical shift vs temperature (K) [1]. The temperature coefficients give structural information as well and have been measured and reported for each amino acid in a solvent exposed, unstructured, flexible random coil conformation [2]. A coefficient which is reduced from this value suggests the amino acid is in a region which is not solvent exposed or, if the temperature coefficient is lowered to a value of 6 ppb/K or less, the NH proton for that amino acid may be in an H bond and more constrained. Here we present temperature coefficients measured experimentally for each amino acid [1], which were then normalized to their random coil temperature coefficients [2] and plotted for Cx26, Cx26G12R and Cx32G12R peptides in Fig. 2. The fractional reduction of the temperature coefficient from its random coil value (which is amino acid dependent) can clearly be seen with the data presented this way. The normalized temperature coefficient plots show that for all 3 N-terminal peptides the NH temperature coefficient is reduced starting from residue 3 and continuing through residue 7 with values reaching less than 80% of the random coil temperature coefficient value. This indicates NH protons in a less solvent exposed region and maybe involved in H bonds. In the NMR structures we see this area is in a helical like turn for the 3 peptide structures and the NH protons are less solvent exposed due to interactions from neighboring hydrophobic side chains. Possible H bonds from the structural analysis are discussed in the NMR study [1]. The NMR structures show larger unconstrained turns within residues 11–15 for Cx26 and Cx26G12R and the normalized temperature coefficients for almost all of the NH protons of these residues are at least 90% of their random coil values with many at the random coil value. This suggests a flexible, unconstrained turn in this region similar to what is shown in the NMR structure [1]. In contrast, the NMR structure shows a tighter helical turn constricted by hydrophobic sidechain interactions within residues 9–13 in Cx32G12R [1]. Examining this region, 9–13, in the normalized temperature coefficient plot for the Cx32G12R peptide the NH protons in the region are very reduced to values of 80% or less of their flexible random coil value. These protons are in a more constrained turn which is less solvent exposed and NMR structural analysis shows a number of H bonds in this area [1]. This data shows that secondary structural propensity analysis using ^13^Cα and ^13^Cβ chemical shifts and normalized temperature coefficients can supplement and support NMR structural data in peptides. It also suggests that valid structural information can be obtained by these methods alone in the case of a disordered peptide or a disordered region of a protein where NMR or x-ray crystallographic information is difficult to obtain.

## Figures and Tables

**Table 1:** Connexin 26 chemical shift assignments.

Residue Name	HN	Hα	Cα	Hβ	Cβ	Hγ	Hδ	Other
1 Met		4.34	56.12	1.81	32.98	2.39		
2 Asp	8.38	4.63	53.75	2.63,2.70	41.16			
3 Trp	8.34	4.55	58.49	3.29, 3.36	29.43		7.296	10.16 (Hε1), 7.50 (Hζ2), 7.24 (HH2), 7.16 (Hζ3), 7.63 (Hε3)
4 Gly	8.52	3.85, 3.93	46.1					
5 Thr	8.01	4.25	63.05	4.240	69.61	1.233		
6. Leu	8.23	4.28	56.2	1.621	42.12	1.687	0.88,0.92	
7. Gln	8.291	4.22	56.6	1.95, 2.04	29.09	2.23		6.83 (Hε1), 7.29 (Hε2)
8. Thr	8.09	4.26	62.97	4.22	69.59	1.20		
9. Ile	8.13	4.13	61.89	1.90	38.67	1.19, 1.51	0.85	0.90 (γCH_3_)
10. Leu	8.38	4.33	55.55	1.585, 1.687	42.12	1.64	0.87, 0.92	
11. Gly	8.37	3.98	45.36					
12. Gly	8.23	3.99	45.27					
13. Val	8.07	4.10	62.55	2.10	32.86	0.94		
14. Asn	8.58	4.71	53.20	2.76, 2.83	38.82		7.00, 7.66	
15. Lys	8.35	4.25	56.23	1.77	32.86	1.34	1.66	2.99 (Hε)
16. His	8.44	4.69	56.09	3.12, 3.21	30.28		7.16	8.09
17. Ser	8.34	4.52	58.40	3.87, 3.92	63.85			
18. Thr	8.35	4.43	61.93	4.32	69.82	1.24		
19. Ser	8.40	4.53	58.35	3.87	63.85			
20. Ile	8.23	4.22	61.51	1.91	38.72	1.22,1.49	0.87	0.94 (γCH_3_)
21. Gly	8.54	3.95	45.35					
22. Lys	7.88	4.20	57.5	1.85	33.00	1.39	1.68	3.00 (Hε2)

**Table 2:** Connexin 26G12R chemical shift assignments.

Residue Name	HN	Hα	Cα	Hβ	Cβ	Hγ	Hδ	Other
1 Met	8.32	4.34	55.45	1.83	33.08	2.41		
2 Asp	8.377	4.64	53.75	2.67,2.70	41.24			
3 Trp	8.40	4.53	58.5	3.31, 3.36	29.46		7.295	10.16 (Hε1), 7.49 (Hζ2), 7.23 (HH2), 7.15 (Hζ3), 7.63 (Hε3)
4 Gly	8.56	3.83,3.93	46.23					
5 Thr	8.02	4.23	63.48	4.20	69.27	1.24		
6. Leu	8.22	4.24	56.5	1.624, 1.674	41.97	1.65	0.87,0.91	
7. Gln	8.276	4.15	56.81	1.94, 2.02	28.91	2.164		6.81 (Hε1), 7.15 (Hε2)
8. Thr	8.02	4.24	63.36	4.25	69.45	1.21		
9. Ile	8.10	4.04	61.88	1.91	38.65	1.19, 1.54	0.85	0.91 (γCH_3_)
10. Leu	8.31	4.27	56.00	1.562, 1.689	42.00	1.75	0.87, 0.90	
11. Gly	8.25	3.94	45.39					
12. Arg	7.99	4.37	56.31	1.79, 1.89	30.85	1.62, 1.67	3.19	
13. Val	8.16	4.09	62.54	2.07	32.77	0.96, 0.92		
14. Asn	8.58	4.73	53.15	2.76, 2.82	38.97		6.99, 7.66	
15. Lys	8.40	4.25	56.58	1.70, 1.77	32.84	1.34	1.64	2.97 (Hε)
16. His	8.38	4.67	56.28	3.10, 3.18	30.80		7.05	7.90
17. Ser	8.30	4.51	58.48	3.92	63.83			
18. Thr	8.34	4.43	61.94	4.32	69.80	1.24		
19. Ser	8.39	4.53	58.5	3.87	63.86			
20. Ile	8.22	4.22	61.55	1.91	38.6	1.22,1.50	0.88	0.94 (γCH_3_)
21. Gly	8.55	3.95	45.38					
22. Lys	7.87	4.20	57.51	1.72, 1.85	33.10	1.39	1.68	3.00 (Hε2)

**Table 3:** Connexin 32G12R chemical shift assignments.

Residue Name	HN	Hα	Cα	Hβ	Cβ	Hγ	Hδ	Other
1 Met	8.35		56.57	1.82	32.70	2.41		
2 Asn	8.46	4.71	53.14	2.74	38.68		6.93, 7.61	
3 Trp	8.25	4.70	57.85	3.26, 3.33	29.52		7.27	10.16 (Hε1), 7.46 (Hζ2), 7.19 (HH2), 7.09 (Hζ3), 7.59 (Hε3)
4 Thr	8.06	4.20	62.74	4.19	69.76	1.12		
5 Gly	7.76	3.77	45.62					
6. Leu	7.94	4.23	56.27	1.49	42.13	1.49	0.83,0.87	
7. Tyr	8.25	4.53	58.47	2.94, 3.10	38.43		7.08	6.79 (Hε1)
8. Thr	8.02	4.20	62.70	4.20	69.72	1.19		
9. Leu	8.11	4.26	56.12	1.65	42.12	1.65	0.88, 0.93	
10. Leu	8.20	4.28	56.23	1.57	42.09	1.68	0.85, 0.91	
11. Ser	8.13	4.36	58.92	3.84, 3.90	63.81			
12. Arg	8.20	4.33	56.58	1.81, 1.91	30.64	1.67	3.174	7.21 (Hε)
13. Val	8.01	4.05	62.80	2.06	32.65	0.94, 0.91		
14. Asn	8.49	4.71	53.15	2.75, 2.84	38.76		6.98, 7.65	
15. Arg	8.33	4.28	56.3	1.72, 1.81	30.66	1.55	3.16	
16. His	8.46	4.71	56.30	3.15, 3.23	30.05		7.16	8.17
17. Ser	8.33	4.51	58.40	3.86, 3.92	63.83			
18. Thr	8.32	4.37	61.92	4.29	69.80	1.24		
19. Ala	8.34	4.37	52.55	1.38	19.25			
20. Ile	8.16	4.17	61.40	1.88	38.72	1.22,1.50	0.88,	0.94 (γCH_3_)
21. Gly	8.53	3.95	45.4					
22. Lys	7.87	4.22	57.24	1.73, 1.87	30.54	1.59	3.19	7.21 (Hε2)
